# Impact of electronic cigarettes (e-cigs) and heat-not-burn/heated tobacco products (HnB/HTP) on asthma and chronic obstructive pulmonary disease: a viewpoint of the Italian Society of Internal Medicine

**DOI:** 10.1007/s11739-024-03648-x

**Published:** 2024-05-28

**Authors:** Paola Andreozzi, Gualberto Gussoni, Giorgio Sesti, Nicola Montano, Antonello Pietrangelo, the Italian Society of Internal Medicine (SIMI) Council Member Group, Stefania Basili, Christian Bracco, Antonio Cittadini, Giovambattista Desideri, Gerardo Mancuso, Marcello Persico, Stafano Petrolani, Mario Pirisi, Leonardo Alberto Sechi, Patrizia Suppressa, Angelo Vacca, Vincenzo Zaccone

**Affiliations:** 1 Predictive Medicine Unit, Department of Internal Medicine, Endocrine-Metabolic Sciences and Infectious Diseases, Azienda Ospedaliero Universitaria Policlinico Umberto I, Rome, Italy; 2 RIDE2Med Foundation, Milan, Italy; 3 Department of Clinical and Molecular Medicine, University of Rome-Sapienza, 00189 Rome, Italy; 4 Department of Clinical Community Sciences, University of Milan, 20122 Milan, Italy; 5 Internal Medicine Unit, Department of Medical and Surgical Sciences, University Hospital of Modena, Modena, Italy

**Keywords:** COPD, Asthma, Smoking cessation, E-cigarettes, Heat-not-burn devices

## Abstract

The association of cigarette smoking with several severe and very severe diseases (oncological, cardiovascular, respiratory) which have dramatic epidemiological, medical, and financial impact, is a well-known public threat. Asthma and chronic obstructive pulmonary disease (COPD) are highly prevalent diseases in Italy, posing significant public health challenges. Tobacco smoking, a primary risk factor for COPD and a common asthma trigger, remains a critical preventable public health issue. While universally acknowledged that quitting smoking drastically reduces the risk of smoking-related health issues, a significant portion of smokers and patients find quitting challenging or undesirable, hence a need for new ways to deal with it. A worth considering alternative might be the switch to electronic cigarettes (e-cig), and heat-not-burn/heated tobacco products (HnB/HTP). Emerging evidence suggests potential benefits in asthma and COPD management when transitioning from traditional smoking to e-cigs or HnB devices. However, the effectiveness of these products in facilitating smoking cessation is still debated, alongside concerns about their role in promoting smoking initiation among non-smokers. Internists are among the physicians who most frequently assist patients with smoking-related diseases, and in this perspective they cannot avoid paying attention to the progressive diffusion of smoking products alternative to the traditional cigarette, and to the controversies with respect to their use. In this context, the Italian Society of Internal Medicine, also recognizing a growing need for clarity for healthcare providers, has undertaken a comprehensive analysis of existing literature to offer an informed perspective on the health impact of e-cigs and HnB/HTP on asthma and COPD.

**Supplementary Information:**

The online version contains supplementary material available at 10.1007/s11739-024-03648-x.

## Introduction

Asthma and chronic obstructive pulmonary disease (COPD) are the most common chronic respiratory diseases globally, representing a major public health challenge and a leading cause of morbidity and mortality [1–4].

The prevalence of COPD varies widely due to differences in survey methods, diagnostic criteria, and analytical approaches [5]. According to the Burden of Obstructive Lung Diseases (BOLD) study, the global prevalence of COPD is estimated at about 10.3% and is expected to rise [6, 7]. In Italy, ISTAT (the National Institute of Statistics) reports a COPD prevalence of 5.6%, although this figure might be underestimated due to random diagnoses and hospitalizations for exacerbations [8].

Global prevalence estimates of asthma indicate about 10% in children and adolescents and 6–7% in adults, varying significantly from 2 to 3% in adults in low-income countries to 10% in high-income countries [9–11]. In Italy, the prevalence of asthma in the population aged over 15 years is about 6.1%, a figure confirmed by the GEIRD study for the age group 20–44 [12, 13]. Recent years show a slight but significant increase in asthma prevalence, especially among children and adolescents [14].

It is important to note that COPD and asthma can overlap in smokers or ex-smokers, making it difficult to differentiate between them in clinical practice due to similarities in symptoms and treatments [3, 4]. Tobacco smoking is a significant environmental risk factor for developing COPD and a common trigger for asthma [3, 4]. The latest survey data from the Istituto Superiore di Sanità-PASSI for 2021–2022 shows that 24.2% of the Italian population are smokers, with the highest prevalence (approximately 29%) among the 18–34 age group [15]. This rate has plateaued after a historical decline (29.8% in 2008, 27.0% in 2014, 24.5% in 2020).

Cigarette smoke is a complex aerosol comprising over 7000 chemical components, each possessing toxic and carcinogenic properties in both its gaseous and particulate phases [16]. These components include nicotine, carbon monoxide, carbon dioxide, heavy metals such as nickel, cadmium, chromium, arsenic, formaldehyde, acrolein, acetone, polycyclic aromatic hydrocarbons (like benzo(a)pyrene), ammonia, and tar [17]. Additionally, cigarette smoke contains a significant amount of reactive oxygen species (ROS) derived from oxygen metabolites that compromise the integrity of physical barriers, leading to increased permeability in respiratory epithelial cells and hindering the clearance of mucus by cilia [18]. Table 1 presents a comprehensive list of the most critical chemical components and toxicants found in tobacco smoke.

**Table 1:** List of tobacco chemical components

Tobacco chemical component	Characteristic and impact on human health
Ammonia	A colorless gas that can be harmful when inhaled in high concentrations. Although the amount of ammonia produced from burning tobacco is relatively small compared to other harmful chemicals, it still contributes to the overall toxicity of tobacco smoke
Carbon Monoxide	A poisonous gas produced by burning tobacco. It reduces the oxygen-carrying capacity of the blood, leading to multiorgan damage and various health risks
Formaldehyde	A colorless gas released when tobacco is burned. It is a recognized carcinogen, linked to an increased risk of lung cancer among other health issues
Heavy metals	Burning tobacco releases toxic heavy metals like lead, arsenic, and cadmium. These metals can cause a range of health problems, including cancer and heart disease
Nicotine	A highly addictive chemical found in tobacco. It can raise blood pressure, increase heart rate, and contribute to the development of cardiovascular diseases
Polycyclic aromatic hydrocarbons (PAHs)	These are a group of chemicals formed when tobacco is burned. PAHs are potent carcinogens and are associated with an increased risk of lung, bladder, and skin cancers
Radioactive elements	Tobacco contains radioactive elements such as polonium-210 and lead-210. These elements can damage DNA and increase the risk of cancer
Tar	A sticky brown substance released in the lungs and airways after smoking tobacco. It causes respiratory problems and contains several cancer-causing chemicals

Globally, about 35–45% of COPD patients are current smokers, and around 20% have never smoked [19]. The proportion of current smokers with asthma aligns with the general population [20], while former smokers range from one quarter to over 40% among asthma patients [21, 22]. Thus, approximately half of the adult asthma population globally are current or former cigarette smokers [23].

Tobacco use, driven by nicotine dependence and behavioral habits, remains a major preventable public health issue. Despite around 70% of smokers wishing to quit, successful long-term cessation often involves multiple attempts: tipically, individuals attempting to quit smoking make about 6 attempts before achieving long-term abstinence. Using a nicotine patch in combination with other nicotine replacement therapy (NRT) products is generally more effective than using a single NRT product alone. As for behavioural support, it can range from brief to intensive and can be effectively delivered in person, or remotely via telephone, text messages, or the internet [24].

Electronic cigarettes have emerged as a potential solution in boosting smoking cessation success rate. However, there is ongoing debate among researchers and public health experts about whether e-cigarettes will significantly enhance smoking cessation efforts, or if they might undermine public health initiatives and potentially increase smoking rates. Another contentious issue is whether e-cigarettes contain fewer and less harmful toxicants compared to conventional combustible cigarettes. Similar claims, regarding their role as an aid in smoking cessation and possessing a less harmful toxicological profile than traditional cigarettes, have also been made for heat-not-burn (HnB) products. A report by Euromonitor International indicated that these products had a retail volume of 233 million units in 2020, accounting for 7% of the overall tobacco market in the country [25]. Additionally, Italy has seen a rise in electronic cigarette use, with an estimated 900,000 users in 2019 [25]. This shift towards alternative smoking methods, coupled with the high prevalence of chronic conditions like COPD and asthma, poses new challenges for the Italian healthcare system. Therefore, a comprehensive understanding of these conditions, their long-term management, and applicable medications is essential. The presence of comorbidities in many of these patients adds complexity to their care [26]. It is crucial to educate patients about self-management, symptom monitoring, adhering to treatments, and lifestyle modifications, including exercise and smoking cessation. Regarding smoking cessation, many patients are curious about the practicality and usefulness of using electronic cigarettes and HnB products as cessation tools.

## Traditional smoking, e-cigarettes, and heat-not-burn tobacco: effects on individuals with asthma and COPD

Asthma and COPD, both characterized as chronic inflammatory airway disorders with limited airflow, share similarities yet differ pathologically due to the types of inflammatory cells involved: COPD is predominantly associated with neutrophils and CD8 lymphocytes, while asthma involves eosinophils and CD4 lymphocytes [27, 28]. Reactive oxygen species (ROS) are known to exacerbate inflammation, contributing to COPD progression, and play a role in worsening asthma symptoms.

Exposure to cigarette smoke activates damage-associated molecular patterns (DAMPs) and pathogen-associated molecular patterns (PAMPs) within lung epithelial cells and alveolar macrophages. This activation stimulates Toll-like receptors (TLR) and NOD-like receptors (NLR), leading to overproduction of ROS and reactive nitric oxide (RNS). Such processes disrupt the balance between oxidation and antioxidants, heightening oxidative stress that damages key lung components like lipids, proteins, nucleic acids, elastin, and collagen. The resulting damage leads to increased apoptosis, impaired skeletal muscle function, excessive mucus production, and reduced steroid receptor efficacy [29, 30]. Cigarette smoking causes not only irreversible DNA mutations but also potentially reversible changes in the epigenetic landscape, including DNA methylation and chromatin modification, accompanied by chronic lung inflammation [31–33]. It can also shift the predominant inflammatory mechanisms in asthma to resemble those in COPD [27].

Long-term tobacco use perpetuates oxidative damage, impairs protective responses and DNA repair, disrupts mitochondrial activity, and affects endoplasmic reticulum homeostasis, thereby exacerbating disease progression [34–38]. Chronic inflammation leads to a continuous influx of inflammatory cells, releasing various mediators including proteases and cytokines, and initiates epithelial–mesenchymal transition (EMT). This constant state induces persistent inflammation and oxidative stress in the lungs, leading to repair and remodelling cycles in both COPD and asthma, promoting mucus hypersecretion, chronic bronchitis, and emphysema [18, 39–41].

Compared to the complex composition of tobacco smoke, e-liquids have a simpler makeup, primarily consisting of vegetable glycerol (VG), propylene glycol (PG), nicotine, and water. The market offers a wide variety of flavored e-liquids, created from synthetic flavor compounds, natural extracts, or a combination of both [42]. The number of compounds in e-cigarette aerosol varies with the flavorings used, sometimes reaching up to 142 [43, 44]. E-cigarettes produce vapor, not smoke. While the inflammatory response may be less intense than with traditional smoking, inhaling heated aerosol can still trigger airway inflammation, possibly leading to respiratory issues. E-cigarette vapor extracts can exhibit acute cytotoxicity, affect cell proliferation, and alter cell morphology, similar to high-nicotine traditional cigarettes. The toxicity varies with different e-liquids used in the same device [45]. Studies have linked the cytotoxic effects of widely used refill fluids to high concentrations of flavor chemicals [46]. Even flavorless e-cigarettes can reduce cell viability and increase pro-inflammatory cytokines, confirming that e-cigarettes' core components independently exert biological effects [47].

E-cigarette aerosol can stimulate ROS production, causing DNA damage, reducing cell viability in a concentration-dependent manner, and impairing phagocytosis. Analyses reveal activation of apoptosis and programmed necrosis pathways [48, 49]. In another study, e-cigarette users showed distinct changes in proteins associated with membrane and mucus formation, increasing susceptibility to respiratory infections [50]. Comparisons between cigarette smokers and e-cigarette users revealed common downregulated genes related to cilia assembly and movement in both groups [51].

However, different outcomes emerged in studies assessing the impact on epithelial barrier integrity, with cigarette smoke extract compromising this integrity, while e-cigarette aerosol did not, suggesting that only cigarette smoke adversely affects host defence mechanisms [52]. E-cigarette aerosol also triggers the release of IL-8/CXCL8 and MMP-9 and increases neutrophil elastase activity, potentially facilitating neutrophil migration to inflammation sites and exacerbating symptoms in asthma and COPD patients [53–55]. The variability in e-liquid composition and device types challenges generalization of these effects. It is important to note that, currently, there are no extensive long-term toxicological or safety studies on vaping conducted in humans.

HnB products produce both primary and secondary emissions containing harmful chemicals such as nicotine, particulate matter, benzene, acrolein, and tobacco-specific nitrosamines. Though these emissions are lower than those from traditional cigarettes, they still pose potential risks [56]. Extended use of HnB products has been associated with reduced endothelial function, increased oxidative stress, and heightened platelet activation [57]. This exposure can affect mitochondrial function, exacerbate airway inflammation and remodeling, increase oxidative stress, and heighten the risk of respiratory infections due to enhanced microbial adherence [58]. Furthermore, studies indicate that HnB products emit more than electronic cigarettes [56]. Comprehensive studies are necessary to fully understand the health implications of both HnB and e-cigarette products.

## Methods

The authors have undertaken a thorough evaluation and analysis of recently published literature, encompassing various studies and reviews, to comprehensively understand the health impacts of e-cigarettes and HnB devices in relation to asthma and COPD. Through this analysis, the Authors seek to shed light on the complex and nuanced effects these smoking alternatives may have on individuals suffering from these respiratory conditions. Specifically, the aim is to offer a well-informed and reasoned perspective on two critical questions:

1. What is the health impact of e-cigarettes and HnB devices on the outcomes of asthma?
2. What is the health impact of e-cigarettes and HnB devices on the outcomes of COPD?

The manuscript is structured as a narrative review, addressing a subject of broad interest, and is grounded in a comprehensive literature review (considering papers published from 2013 to 2023) conducted primarily through PubMed. Relevant keywords including MeSH terms were meticulously defined through a collaborative effort by all co-authors, ensuring the inclusion of pertinent studies. This process entailed multiple iterations to accurately encompass the desired range of studies. These studies were essential for extracting key informative evidence, which was then used to formulate the Discussion and Conclusions sections.

Beyond the development of search criteria, a significant aspect of the manuscript's creation was the consensus-building process among the co-authors. This step is critical in scholarly writing, as it guarantees the manuscript's findings, and interpretations accurately represent the collective understanding of the entire research team, thereby reducing the risk of inaccuracies or misinterpretations. This phase entailed an exhaustive review and discussion of the relevant literature until there was unanimous agreement among all co-authors on the interpretation and phrasing of the key findings.

References were thoroughly analysed, and the most informative findings/evidence from this analysis were organized and summarized into three distinct tables. The tables report the most relevant literature articles selected by the working group that provide the informative key evidence on the health impact of e-cigarettes/ HnB on general health status (supplementary Table 2), and on asthma (supplementary Table 3) and COPD (supplementary Table 4) clinical outcomes.

## Results

### Effects on health status

This section concerns the analysis of the literature regarding general characteristics of e-cig and HnB tobacco products, and potential effects on health status.

The evidence referable to this section, listed in supplementary Table 2, reflects data from the literature which may in some cases appear contradictory. These can be summarized in a position which, on the one hand, indicates that alternative products to traditional tobacco contain toxic substances capable to express harmful effects (evidences 1–5 and 7) on the various systems (respiratory and others) and through multiple mechanisms, on the other hand acknowledges that e-cigs and HnB tobacco products do not contain some of the most harmful toxicants produced by smoke from combustion cigarettes (evidences 6 and 8).

In the specific field of respiratory diseases, e-cigs in particular can express harmful effects related to nicotine and/or flavoured e-liquids which are expressed through mechanisms of cytotoxicity, oxidative stress, inflammation, airway hyper-reactivity, airway remodelling, mucin production, apoptosis and emphysematous changes (evidences 3 and 4), as well as to increased susceptibility to viral infection through an increase in lung ACE2 expression, a mechanism that we have well known with SARS-CoV-2 infection (evidence 5). For its part, exposure to HnB products has been reported to alter mitochondrial function, which may exaggerate airway inflammation and remodelling (evidence 7).

On the other hand, exclusive e-cigarette and HnB products users have lower risk of exposure to tobacco smoke toxicants and carcinogens compared to cigarette smokers (evidences 6 and 8) and consequently undergo less significant pulmonary changes (evidence 9).

Finally, we should not forget the potential harm, nicotine-dependent, and demonstrated in murine models, which also affects the use in pregnancy of smoking products alternative to traditional cigarettes (evidence 10).

### Effects on asthma clinical outcomes

In the specific field of asthma, epidemiological studies have documented that among e-cigarettes users there is an increased incidence of the disease compared to non-users (evidence 1, supplementary Table 3). This finding is important considering the huge number of e-cig users among young people who have never smoked. It is known that common flavouring agents are recognized as primary irritants of mucosal tissue of respiratory tract, and the thermal decomposition of propylene glycol and vegetable glycerine (the base constituents of e-liquids) may produce reactive carbonyls, which have known respiratory toxicities. Therefore, it is not surprising that in subjects affected by asthma, the use of e-cigarettes is associated with worse symptomatology and impaired lung function (evidence 2). However, lower odds of negative outcomes have been reported when there is a switch from traditional cigarettes to e-cigs (evidence 3), and this allow to consider that these products can be an option for asthmatic patients who cannot quit smoking by other methods. Heterogeneous findings are available in the literature on the role of e-cigarettes in helping asthmatic subjects to quit smoking (evidence 4).

However, as an overall consideration, due to the relatively recent use of e-cigarette (and HnB as well) and the low number of experimental data and consistent evidence, it is hard to draw conclusive indications regarding toxicological and clinical consequences of the use of e-cigarettes (and more generally, of alternative tobacco products) (evidence 5).

### Effects on COPD clinical outcomes

From a qualitative point of view, the considerations expressed for alternative products to traditional tobacco with reference to asthma, are reproducible in the context of the COPD. In particular, also for COPD a higher incidence of the disease is observed in e-cigarettes users compared to non-users (evidence 1, supplementary Table 4) and e-cigarette use leads to an increase of symptoms and impaired lung function in COPD patients (evidence 2). However, e-cigarettes users have been reported to have less negative outcomes if compared to combustible cigarettes or dual smoking (combustible + electronic cigarettes) (evidence 3); further, in the case of HnB products, their use after switch from combustible cigarettes seems associated with reduced exacerbations, improvements in symptomatology and activity level in COPD patients (evidence 4). Inconclusive findings are available on the possible role of e-cigarettes (and HnB products) in helping to reduce or stop smoking in COPD patients (evidence 5). Also in the case of COPD, to express overall considerations on the impact of products alternative to combustible cigarettes is a challenging effort, due to a poor availability of methodologically solid experimental findings, and the relatively recent use of these products combined with the need of decades of chronic smoking for development of COPD (evidence 6).

## Discussion

The well-established connection between cigarette smoking and various severe diseases, including cancer, cardiovascular, and respiratory illnesses, represents a significant public health concern due to its epidemiological, medical, and financial impacts. Internists, who frequently encounter patients with smoking-related diseases, must stay informed about the emerging trends in smoking alternatives such as e-cigarettes and HnB products, along with the ongoing scientific debate surrounding their use. This aligns with the commitment of the Italian Scientific Society of Internal Medicine (SIMI), to analyze the available scientific literature on these products, especially focusing on chronic respiratory diseases like asthma and COPD.

While it might seem obvious, a key principle in addressing cigarette smoking is the imperative to prevent the initiation of smoking habits and to encourage cessation. This is particularly crucial for individuals with chronic diseases. For asthma and COPD patients, smoking cessation is known to improve symptoms and slow down the decline in lung function. However, physicians often observe that many of these patients, despite their intentions, do not respond well to conventional smoking cessation interventions, including behavioural support and licensed therapies like nicotine replacement therapy (NRT) and medications, or achieve only limited success. Consequently, many continue to smoke despite experiencing adverse symptoms [59].

In the past decade or so, there has been considerable debate within the scientific community about whether new alternatives to traditional combustion cigarettes, particularly e-cigarettes and HnB products, offer reduced toxicity levels and can be effective tools for quitting or reducing traditional cigarette smoking. Despite the heterogeneity of available data and the challenges in extrapolating results from cellular or animal models to human exposure, the evidence suggests that these alternative products are not risk-free. Their use, compared to not smoking, increases the risk of asthma and/or COPD, exacerbating symptoms and impairing lung function.

Although the use of e-cigarettes or HnB products is not without harm and their long-term effects remain uncertain, independent reviews and expert opinions have concluded that these alternatives are likely less harmful than traditional smoking. Studies involving humans have shown that these products, when used by smokers, are associated with significant reductions in blood or urinary biomarkers of tobacco toxicants, particularly when fully switching, and to some extent in case of dual use. While the extent to which these biomarkers represent potential lung toxicity is not entirely clear, several studies have indicated that former smokers who switch to e-cigarettes or HnB products tend to experience lower odds of respiratory outcomes, fewer exacerbations, and improvements in symptoms and physical activity compared to those who continue smoking traditional cigarettes or engage in dual use. Given these findings, the potential of these products as harm reduction options for individuals unable or unwilling to quit smoking merits consideration, especially considering the low long-term cessation rates achieved with pharmacological and behavioural treatments.

One hypothesis is that e-cigarettes and HnB products might support smoking reduction or cessation due to better nicotine release, decreasing nicotine concentration overtime, and mimicking the behavioural and sensory aspects of cigarette smoking. Recent publications, including a commentary in Nature [60] and a comprehensive review [59], have reported higher smoking cessation rates in individuals using nicotine electronic cigarettes compared to those using nicotine replacement therapy. These findings align with what has been previously reported in systematic reviews and randomized controlled and population studies [61–65]. However, the current data are still insufficient to support unanimous recommendations in this regard [66, 67].

There is a growing public health concern about the increasing use of e-cigarettes and HnB products among never-smokers, particularly adolescents and young people, which may lead to nicotine addiction and potentially increase the likelihood of future conventional smoking [68–76]. Ironically, while e-cigarettes were introduced as an aid for smoking cessation in adult smokers, epidemiological data indicate that these products often serve as a gateway to tobacco initiation among tobacco-naive adolescents. Factors such as product design, flavours, perceived safety, and targeted marketing strategies on media and social networks have heightened the appeal of e-cigarettes (and HnB products) to younger populations [77]. Recent reports in the United States have shown declines in dual usage rates among youth, offering less concerning trends in this regard [78, 79], but concerted efforts are still needed to address this public health threat, such as including warnings on product packaging about adverse effects and the risk of nicotine addiction [80], and promoting awareness campaigns through new media channels. The aforementioned article in Nature asserts that "E-cigarettes are not a panacea for the harms caused by cigarette smoking, but they can contribute to this public health goal. However, the endorsement of e-cigarettes for smoking cessation is likely contingent on continued efforts to restrict access and use by young non-smokers. These two objectives should coexist" [60].

Furthermore, it is crucial for healthcare professionals to receive education, training, and support to improve counselling for patients and the public about the use of tobacco products, their potential health risks, behaviours change, and various cessation options [81, 82]. Smokers with asthma or COPD have compelling reasons to quit or at least reduce cigarette consumption and the associated harms. Clinicians should actively explore all available methods to assist their patients in achieving these goals but should prioritize recommending evidence-based treatments.

Given the relatively recent advent of vaping and HnB cigarettes compared to the long duration of chronic smoking required for the development of diseases like COPD, the evolving landscape of these products, and the scarcity of experimental data and consistent evidence, it is not surprising that it is currently difficult to draw definitive conclusions regarding the toxicological, clinical, and public health impact of these alternatives to traditional combustible cigarettes. In this context, high-quality and independent studies with adequate sample sizes and longer follow-up periods are necessary to better document the effects of these products.

## Conclusions

Tobacco smoking continues to be a major cause of mortality worldwide. Efforts to promote smoking cessation and prevent smoking initiation are crucial. Alternative products to traditional cigarettes tend to produce fewer toxic substances, however they are not risk-free and their use increases the risk of asthma or COPD compared to non-smokers, despite variations in available data and interpretation [83, 84]. Switching from traditional cigarettes to these alternatives can lead to better health outcomes [60]. Although recent data suggest a potential for these combustion-free products to aid in smoking cessation or reduction compared to traditional methods, their potential role in promoting smoking initiation is still up for dispute. High-quality studies with larger sample sizes and longer follow-up periods are essential to thoroughly understand the impact of exposure to alternative tobacco products. Establishing a robust support system for healthcare professionals to provide effective counselling on smoking, including traditional cigarettes, e-cigarettes, and HnB products, is imperative.

## Supplementary Information

Below is the link to the electronic supplementary material.

Supplementary file1 (DOCX 242 KB)

## Data Availability

Not Applicable.
