# Supplementary material for: Impact of electronic cigarettes (e-cigs) and heat-not-burn/heated tobacco products (HnB/HTP) on asthma and chronic obstructive pulmonary disease: a viewpoint of the Italian Society of Internal Medicine
Source: Intern Emerg Med. 2024 May 28;19(7):1829–37. doi: 10.1007/s11739-024-03648-x (PMC11467123; doi:10.1007/s11739-024-03648-x)
Supplement: Supplementary file 1 — Supplementary file1 (DOCX 242 KB) [file 11739_2024_3648_MOESM1_ESM.docx]

**Supplementary Table 2. The general health impact of e-cigarettes/ HnB. Recent studies (2014-2023) providing the listed Key informative evidence.**

|  | **Key informative evidence** | **Most relevant supporting articles** |
| --- | --- | --- |
| 1 | Although in lower concentrations, e-cigarette aerosols contain many harmful chemicals found in tobacco smoke, such as nicotine, formaldehyde, and acetaldehyde. | Kaur G 2018[1]  Kaur G 2020 [2] |
| 2 | Unique components of flavored e-cigarettes, including propylene glycol and vegetable glycerin (the primary constituents of e-liquids), along with various flavoring chemicals like vanillin, chocolate, and menthol, are likely to cause respiratory effects and toxicities. | Clapp P 2017 [3]  Son Y 2019 [4]  Smith M 2021 [5] |
| 3 | Respiratory disease outcomes associated with e-cigarettes, linked to nicotine and/or flavored e-liquids, are influenced by multiple mechanisms including cytotoxicity, oxidative stress, inflammation, airway hyperreactivity, airway remodeling, mucin production, apoptosis, and emphysematous changes. | Sussan T 2015 [6]  Higham A 2016 [7]  Shivalingappa P 2016 [8]  Garcia-Arcos I 2016 [9]  Dhand R 2017 [10]  Jankwoski M 2017 [11]  Palamidas A 2017 [12]  Rowell T 2017 [13]  Higham A 2018 [14]  Ghosh A 2018 [15]  Reidel B 2018 [16]  Bozier J 2019 [17]  Ghosh A 2019 [18]  Gilpin D 2019 [19]  Lin V 2019 [20]  Madison M 2019 [21]  Son Y 2019 [4]  Gellatly S 2020 [22]  Kaur G 2020 [2]  Rosenkilde Laursen K 2020 [23]  Tsai M 2020 [24]  Grondin C 2021 [25]  Sinha I 2022 [26] |
| 4 | Vaping has been observed to trigger inflammatory responses similar to those caused by tobacco smoke. | Lappas A 2018 [27]  Kaur G 2018 [1]  Reidel B 2018 [16]  Taha H 2020 [28]  Kotoulas S 2020 [29]  Traboulsi H 2020 [30]  Kotoulas S 2021 [31] |
| 5 | Vaping may increase lung ACE2 expression, potentially leading to a heightened susceptibility to viral infections and the progression of severe respiratory distress or diseases. | McAlinden K 2021 [32] |
| 6 | The content of toxic chemicals in Heat-not-Burn (HnB) products aerosol appears to be lower than in traditional cigarette smoke. However, a number of harmful and potentially harmful constituents have been reported to be higher in HnB aerosol than in cigarette smoke | Basaran R 2019 [33]  Phillips B 2019 [34]  Znyk M 2021 [35]  Upadhyay S 2023 [36]  Hashizume T 2023 [37] |
| 7 | In preclinical models exposure to HnB products has been shown to affect mitochondrial function and to increase levels of some cytokines, potentially exacerbating airway inflammation and remodeling, causing acute stress responses in the lung and affecting small airway function | Znyk M 2021 [35]  Sawa M 2022 [38]  Koike S 2022 [39]  Gu J 2023 [40]  Goebel I 2023 [41] |
| 8 | Exclusive users of e-cigarettes face a lower risk of exposure to tobacco smoke toxicants and carcinogens compared to traditional cigarette smokers. | Ratajczak A 2018 [42]  Hernandez M 2021 [43]  Wills T 2021 [44]  Wills T 2022 [45] |
| 9 | E-cigarettes, due to reduced exposure to toxicants, cause less significant pulmonary changes than those induced by cigarette smoke. | Van Staden S 2013 [46]  Dhand R 2017 [10]  Jankwoski M 2017 [11]  Madison M 2019 [21]  Lavrynenko O 2020 [47]  Czekala L 2021 [48]  Rayner R 2021 [49]  Wills T 2021 [44]  Wong E 2021 [50] |
| 10 | Prenatal exposure to nicotine presents significant toxicity risks; thus, the potential harm of using electronic cigarettes during pregnancy is a substantial concern. | Suter M 2015 [51]  Spindel E 2016 [52]  Clapp P 2017 [3]  McEvoy C 2017 [53]  Zakarya R 2019 [54]  Collaco J 2022 [55]  Orzabal M 2022 [56] |

**REFERENCES**

1. Kaur G, Pinkston R, McLemore B, Dorsey WC, Batra S (2018) Immunological and toxicological risk assessment of e-cigarettes. Eur Respir Rev. https://doi.org/10.1183/16000617.0119-2017

2. Kaur G, Singh K, Maremanda KP, Li D, Chand HS, Rahman I (2020) Differential plasma exosomal long non-coding RNAs expression profiles and their emerging role in E-cigarette users, cigarette, waterpipe, and dual smokers. PLoS One. https://doi.org/10.1371/JOURNAL.PONE.0243065

3. Clapp PW, Jaspers I (2017) Electronic Cigarettes: Their Constituents and Potential Links to Asthma. Curr Allergy Asthma Rep. https://doi.org/10.1007/S11882-017-0747-5

4. Son Y, Mishin V, Laskin JD, Mainelis G, Wackowski OA, Delnevo C, Schwander S, Khlystov A, Samburova V, Meng Q (2019) Hydroxyl Radicals in E-Cigarette Vapor and E-Vapor Oxidative Potentials under Different Vaping Patterns. Chem Res Toxicol 32:1087–1095

5. Smith MR, Jarrell ZR, Orr M, Liu KH, Go YM, Jones DP (2021) Metabolome-wide association study of flavorant vanillin exposure in bronchial epithelial cells reveals disease-related perturbations in metabolism. Environ Int. https://doi.org/10.1016/J.ENVINT.2020.106323

6. Sussan TE, Gajghate S, Thimmulappa RK, et al (2015) Exposure to electronic cigarettes impairs pulmonary anti-bacterial and anti-viral defenses in a mouse model. PLoS One. https://doi.org/10.1371/JOURNAL.PONE.0116861

7. Higham A, Rattray NJW, Dewhurst JA, Trivedi DK, Fowler SJ, Goodacre R, Singh D (2016) Electronic cigarette exposure triggers neutrophil inflammatory responses. Respir Res. https://doi.org/10.1186/S12931-016-0368-X

8. Shivalingappa PC, Hole R, Van Westphal C, Vij N (2016) Airway Exposure to E-Cigarette Vapors Impairs Autophagy and Induces Aggresome Formation. Antioxid Redox Signal 24:186–204

9. Garcia-Arcos I, Geraghty P, Baumlin N, et al (2016) Chronic electronic cigarette exposure in mice induces features of COPD in a nicotine-dependent manner. Thorax 71:1119–1129

10. Dhand R (2017) Inhaled Drug Therapy 2016: The Year in Review. Respir Care 62:978–996

11. Jankowski M, Brozek G, Lawson J, Skoczyński S, Zejda JE (2017) E-smoking: Emerging public health problem? Int J Occup Med Environ Health 30:329–344

12. Palamidas A, Tsikrika S, Katsaounou PA, Vakali S, Gennimata SA, kaltsakas G, Gratziou C, Koulouris N (2017) Acute effects of short term use of ecigarettes on Airways Physiology and Respiratory Symptoms in Smokers with and without Airway Obstructive Diseases and in Healthy non smokers. Tob Prev Cessat. https://doi.org/10.18332/TPC/67799

13. Rowell TR, Reeber SL, Lee SL, Harris RA, Nethery RC, Herring AH, Glish GL, Tarran R (2017) Flavored e-cigarette liquids reduce proliferation and viability in the CALU3 airway epithelial cell line. Am J Physiol Lung Cell Mol Physiol 313:L52–L66

14. Higham A, Bostock D, Booth G, Dungwa J V., Singh D (2018) The effect of electronic cigarette and tobacco smoke exposure on COPD bronchial epithelial cell inflammatory responses. Int J Chron Obstruct Pulmon Dis 13:989–1000

15. Ghosh A, Coakley RC, Mascenik T, et al (2018) Chronic E-Cigarette Exposure Alters the Human Bronchial Epithelial Proteome. Am J Respir Crit Care Med 198:67–76

16. Reidel B, Radicioni G, Clapp PW, Ford AA, Abdelwahab S, Rebuli ME, Haridass P, Alexis NE, Jaspers I, Kesimer M (2018) E-Cigarette Use Causes a Unique Innate Immune Response in the Lung, Involving Increased Neutrophilic Activation and Altered Mucin Secretion. Am J Respir Crit Care Med 197:492–501

17. Bozier J, Rutting S, Xenaki D, Peters M, Adcock I, Oliver BG (2019) Heightened response to e-cigarettes in COPD. ERJ Open Res. https://doi.org/10.1183/23120541.00192-2018

18. Ghosh A, Coakley RD, Ghio AJ, Muhlebach MS, Esther CR, Alexis NE, Tarran R (2019) Chronic E-Cigarette Use Increases Neutrophil Elastase and Matrix Metalloprotease Levels in the Lung. Am J Respir Crit Care Med 200:1392–1401

19. Gilpin DF, McGown KA, Gallagher K, Bengoechea J, Dumigan A, Einarsson G, Elborn JS, Tunney MM (2019) Electronic cigarette vapour increases virulence and inflammatory potential of respiratory pathogens. Respir Res. https://doi.org/10.1186/S12931-019-1206-8

20. Lin VY, Fain MD, Jackson PL, Berryhill TF, Wilson LS, Mazur M, Barnes SJ, Edwin Blalock J, Vamsee Raju S, Rowe SM (2019) Vaporized E-Cigarette Liquids Induce Ion Transport Dysfunction in Airway Epithelia. Am J Respir Cell Mol Biol 61:162–173

21. Madison MC, Landers CT, Gu BH, et al (2019) Electronic cigarettes disrupt lung lipid homeostasis and innate immunity independent of nicotine. J Clin Invest 129:4290–4304

22. Gellatly S, Pavelka N, Crue T, et al (2020) Nicotine-Free e-Cigarette Vapor Exposure Stimulates IL6 and Mucin Production in Human Primary Small Airway Epithelial Cells. J Inflamm Res 13:175–185

23. Rosenkilde Laursen K, Bønløkke JH, Bendstrup E, et al (2020) An RCT of acute health effects in COPD-patients after passive vape exposure from e-cigarettes. Eur Clin Respir J. https://doi.org/10.1080/20018525.2020.1861580

24. Tsai MC, Byun MK, Shin J, Crotty Alexander LE (2020) Effects of e-cigarettes and vaping devices on cardiac and pulmonary physiology. J Physiol 598:5039–5062

25. Grondin CJ, Davis AP, Wiegers JA, Wiegers TC, Sciaky D, Johnson RJ, Mattingly CJ (2021) Predicting molecular mechanisms, pathways, and health outcomes induced by Juul e-cigarette aerosol chemicals using the Comparative Toxicogenomics Database. Curr Res Toxicol 2:272–281

26. Sinha I, Goel R, Bitzer ZT, Trushin N, Liao J, Sinha R (2022) Evaluating electronic cigarette cytotoxicity and inflammatory responses in vitro. Tob Induc Dis. https://doi.org/10.18332/TID/147200

27. Lappas AS, Tzortzi AS, Konstantinidi EM, Teloniatis SI, Tzavara CK, Gennimata SA, Koulouris NG, Behrakis PK (2018) Short-term respiratory effects of e-cigarettes in healthy individuals and smokers with asthma. Respirology 23:291–297

28. Taha HR, Al-Sawalha NA, Alzoubi KH, Khabour OF (2020) Effect of E-Cigarette aerosol exposure on airway inflammation in a murine model of asthma. Inhal Toxicol 32:503–511

29. Kotoulas SC, Pataka A, Domvri K, et al (2020) Acute effects of e-cigarette vaping on pulmonary function and airway inflammation in healthy individuals and in patients with asthma. Respirology 25:1037–1045

30. Traboulsi H, Cherian M, Rjeili MA, Preteroti M, Bourbeau J, Smith BM, Eidelman DH, Baglole CJ (2020) Inhalation Toxicology of Vaping Products and Implications for Pulmonary Health. Int J Mol Sci. https://doi.org/10.3390/IJMS21103495

31. Kotoulas SC, Katsaounou P, Riha R, Grigoriou I, Papakosta D, Spyratos D, Porpodis K, Domvri K, Pataka A (2021) Electronic Cigarettes and Asthma: What Do We Know So Far? J Pers Med. https://doi.org/10.3390/JPM11080723

32. McAlinden KD, Lu W, Ferdowsi PV, et al (2021) Electronic Cigarette Aerosol Is Cytotoxic and Increases ACE2 Expression on Human Airway Epithelial Cells: Implications for SARS-CoV-2 (COVID-19). J Clin Med 10:1–18

33. Başaran R, Güven NM, Eke BC (2019) An Overview of iQOS® as a New Heat-Not-Burn Tobacco Product and Its Potential Effects on Human Health and the Environment. Turk J Pharm Sci 16:371–374

34. Phillips B, Szostak J, Titz B, et al (2019) A six-month systems toxicology inhalation/cessation study in ApoE-/- mice to investigate cardiovascular and respiratory exposure effects of modified risk tobacco products, CHTP 1.2 and THS 2.2, compared with conventional cigarettes. Food Chem Toxicol 126:113–141

35. Znyk M, Jurewicz J, Kaleta D (2021) Exposure to Heated Tobacco Products and Adverse Health Effects, a Systematic Review. Int J Environ Res Public Health. <https://doi.org/10.3390/IJERPH18126651>

36. Upadhyay S, Rahman M, Johanson G et al (2023) Heated Tobacco Products: insights into composition and toxicity. Toxics 11:667

37. Hashizume T, Ishikawa S, Matsumura K et al (2023) Chemical and in vitro toxicological comparison of emissions from a heated tobacco product and the 1R6F reference cigarette. Toxicol Rep 10:281-292

38. Koike S, Sato K, Sawa M et al (2022) Exposure to Heated Tobacco Products aerosol causes acute stress responses in the lung of mouse. Antioxidants (Basel) 11:2329

39. Sawa M, Ushiyama A, Inaba Y et al (2022) Increased oxidative stress and effects on inflammatory cytokine secretion by heated tobacco products aerosol exposure to mice. Biochem Biophys Res Commun 610:43-48

40. Gu J, Gong D, Wang Y et al (2023) Chronic exposure to IQOS results in impaired pulmonary function and lung tissue damage in mice. Toxicol Lett 374:1-10

41. Goebel I, Mohr T, Axt PN et al (2023) Impact of Heated Tobacco Products, E-cigarettes, and combustible cigarettes on small airways and arterial stiffness. Toxics 11:758

42. Ratajczak A, Feleszko W, Smith DM, Goniewicz M (2018) How close are we to definitively identifying the respiratory health effects of e-cigarettes? Expert Rev Respir Med 12:549–556

43. Hernandez ML, Burbank AJ, Alexis NE, Rebuli ME, Hickman ED, Jaspers I, Guidos G (2021) Electronic Cigarettes and Their Impact on Allergic Respiratory Diseases: A Work Group Report of the AAAAI Environmental Exposures and Respiratory Health Committee. J Allergy Clin Immunol Pract 9:1142–1151

44. Wills TA, Soneji SS, Choi K, Jaspers I, Tam EK (2021) E-cigarette use and respiratory disorders: an integrative review of converging evidence from epidemiological and laboratory studies. Eur Respir J. https://doi.org/10.1183/13993003.01815-2019

45. Wills TA, Choi K, Pokhrel P, Pagano I (2022) Tests for confounding with cigarette smoking in the association of E-cigarette use with respiratory disorder: 2020 National-Sample Data. Prev Med (Baltim). https://doi.org/10.1016/J.YPMED.2022.107137

46. van Staden SR, Groenewald M, Engelbrecht R, Becker PJ, Hazelhurst LT (2013) Carboxyhaemoglobin levels, health and lifestyle perceptions in smokers converting from tobacco cigarettes to electronic cigarettes. S Afr Med J 103:865–868

47. Lavrynenko O, Titz B, Dijon S, et al (2020) Ceramide ratios are affected by cigarette smoke but not heat-not-burn or e-vapor aerosols across four independent mouse studies. Life Sci. https://doi.org/10.1016/J.LFS.2020.118753

48. Czekala L, Wieczorek R, Simms L, et al (2021) Multi-endpoint analysis of human 3D airway epithelium following repeated exposure to whole electronic vapor product aerosol or cigarette smoke. Curr Res Toxicol 2:99–115

49. Rayner RE, Makena P, Prasad GL, Cormet-Boyaka E (2021) Cigarette smoke preparations, not electronic nicotine delivery system preparations, induce features of lung disease in a 3D lung repeat-dose model. Am J Physiol Lung Cell Mol Physiol 320:L276–L287

50. Wong ET, Szostak J, Titz B, et al (2021) A 6-month inhalation toxicology study in Apoe-/- mice demonstrates substantially lower effects of e-vapor aerosol compared with cigarette smoke in the respiratory tract. Arch Toxicol 95:1805–1829

51. Suter MA, Mastrobattista J, Sachs M, Aagaard K (2015) Is there evidence for potential harm of electronic cigarette use in pregnancy? Birth Defects Res A Clin Mol Teratol 103:186–195

52. Spindel ER, McEvoy CT (2016) The Role of Nicotine in the Effects of Maternal Smoking during Pregnancy on Lung Development and Childhood Respiratory Disease. Implications for Dangers of E-Cigarettes. Am J Respir Crit Care Med 193:486–494

53. McEvoy CT, Spindel ER (2017) Pulmonary Effects of Maternal Smoking on the Fetus and Child: Effects on Lung Development, Respiratory Morbidities, and Life Long Lung Health. Paediatr Respir Rev 21:27–33

54. Zakarya R, Adcock I, Oliver BG (2019) Epigenetic impacts of maternal tobacco and e-vapour exposure on the offspring lung. Clin Epigenetics. https://doi.org/10.1186/S13148-019-0631-3

55. Collaco JM, McGrath-Morrow SA (2022) Developmental Effects of Electronic Cigarette Use. Compr Physiol 12:3337–3346

56. Orzabal MR, Naik VD, Lee J, Hillhouse AE, Brashear WA, Threadgill DW, Ramadoss J (2022) Impact of E-cig aerosol vaping on fetal and neonatal respiratory development and function. Transl Res 246:102–114

**Supplementary Table 3. The health impact of e-cigarettes/ HnB on asthma outcomes. Recent studies (2014-2023) providing the listed Key informative evidence.**

|  | **Key informative evidence** | **Most relevant supporting articles** |
| --- | --- | --- |
| 1 | E-cigarette users have been observed to have a higher incidence of asthma compared to those who do not use e-cigarettes. | Clapp P 2017 [1]  Kim S 2017 [2]  Schweitzer R 2017 [3]  Lee A 2019 [4]  Osei A 2019 [5]  Wills T 2019 [6]  Bhatta D 2020 [7]  Parekh T 2020 [8]  Xie W 2020 [9]  Wills T 2020 [10]  Bircan E 2021 [11]  Han C 2021 [12]  Kotoulas S 2021 [13]  Xian S 2021 [14]  Walker C 2021 [15]  Wills T 2021 [16]  Gugala E 2022 [17]  Li X 2022 [18]  Wills T 2022 [19] |
| 2 | In individuals with asthma, the use of e-cigarettes has been associated with an exacerbation of symptoms and a decline in lung function. | Palamidas A 2017 [20]  Hedman L 2018 [21]  Wang J 2018 [22]  Bayly J 2019 [23]  Thirion Romero I 2019 [24]  Amato L 2020 [25]  Underner M 2020 [26]  Chaffee B 2021 [27]  Entwistle M 2021 [28]  Kotoulas S 2021 [13]  Gugala E 2022 [17] |
| 3 | Former smokers with asthma who have switched to e-cigarettes demonstrate a lower likelihood of experiencing adverse respiratory outcomes. | Polosa R 2014 [29]  Goniewicz M 2020 [30]  Solinas A 2020 [31] |
| 4 | Despite increasing evidence, there is no unanimous agreement among experts about the effectiveness of e-cigarettes in assisting asthmatic adults in quitting smoking. | Franks A 2018 [32]  Hedman L 2018 [21]  Bhatnagar A 2019 [33]  Underner M 2020 [26]  Hernandez M 2021 [34]  Kotoulas SC 2021 [13] |
| 5 | Given the relatively recent emergence of vaping, the constantly changing nature of e-cigarettes, and the lack of extensive experimental data and consistent findings, it is currently challenging to definitively determine the toxicological, immunological, and clinical impacts of e-cigarette aerosols. | Kaur G 2018 [35]  Bozier J 2020 [36]  Gordon T 2022 [37] |

**REFERENCES**

1. Clapp PW, Jaspers I (2017) Electronic Cigarettes: Their Constituents and Potential Links to Asthma. Curr Allergy Asthma Rep. https://doi.org/10.1007/S11882-017-0747-5

2. Kim SY, Sim S, Choi HG (2017) Active, passive, and electronic cigarette smoking is associated with asthma in adolescents. Sci Rep. https://doi.org/10.1038/S41598-017-17958-Y

3. Schweitzer RJ, Wills TA, Tam E, Pagano I, Choi K (2017) E-cigarette use and asthma in a multiethnic sample of adolescents. Prev Med (Baltim) 105:226–231

4. Lee A, Lee SY, Lee KS (2019) The Use of Heated Tobacco Products is Associated with Asthma, Allergic Rhinitis, and Atopic Dermatitis in Korean Adolescents. Sci Rep. https://doi.org/10.1038/S41598-019-54102-4

5. Osei AD, Mirbolouk M, Orimoloye OA, Dzaye O, Uddin SMI, Dardari ZA, Defilippis AP, Bhatnagar A, Blaha MJ (2019) The association between e-cigarette use and asthma among never combustible cigarette smokers: behavioral risk factor surveillance system (BRFSS) 2016 & 2017. BMC Pulm Med. https://doi.org/10.1186/S12890-019-0950-3

6. Wills TA, Pagano I, Williams RJ, Tam EK (2019) E-cigarette use and respiratory disorder in an adult sample. Drug Alcohol Depend. https://doi.org/10.1016/j.drugalcdep.2018.10.004

7. Bhatta DN, Glantz SA (2020) Association of E-Cigarette Use With Respiratory Disease Among Adults: A Longitudinal Analysis. Am J Prev Med 58:182–190

8. Parekh T, Owens C, Fay K, Phillips J, Kitsantas P (2020) Use of e-Cigarettes and Development of Respiratory Conditions in Women of Childbearing Age. South Med J 113:488–494

9. Xie W, Kathuria H, Galiatsatos P, Blaha MJ, Hamburg NM, Robertson RM, Bhatnagar A, Benjamin EJ, Stokes AC (2020) Association of Electronic Cigarette Use With Incident Respiratory Conditions Among US Adults From 2013 to 2018. JAMA Netw Open. https://doi.org/10.1001/JAMANETWORKOPEN.2020.20816

10. Wills TA, Choi K, Pagano I (2020) E-Cigarette Use Associated With Asthma Independent of Cigarette Smoking and Marijuana in a 2017 National Sample of Adolescents. J Adolesc Health 67:524–530

11. Bircan E, Bezirhan U, Porter A, Fagan P, Orloff M (2021) Electronic cigarette use and its association with asthma, chronic obstructive pulmonary disease (COPD) and asthma-COPD overlap syndrome among never cigarette smokers. Tob Induc Dis 19:1–10

12. Han CH, Chung JH (2021) Factors associated with electronic cigarette use among adolescents asthma in the Republic of Korea. J Asthma 58:1451–1459

13. Kotoulas SC, Katsaounou P, Riha R, Grigoriou I, Papakosta D, Spyratos D, Porpodis K, Domvri K, Pataka A (2021) Electronic Cigarettes and Asthma: What Do We Know So Far? J Pers Med. https://doi.org/10.3390/JPM11080723

14. Xian S, Chen Y (2021) E-cigarette users are associated with asthma disease: A meta-analysis. Clin Respir J 15:457–466

15. Walker CJ, Christian WJ (2021) Estimating the Population Attributable Fraction of Asthma Due to Electronic Cigarette Use and Other Risk Factors Using Kentucky Behavioral Risk Factor Survey Data, 2016-2017. Subst Use Misuse 56:353–358

16. Wills TA, Soneji SS, Choi K, Jaspers I, Tam EK (2021) E-cigarette use and respiratory disorders: an integrative review of converging evidence from epidemiological and laboratory studies. Eur Respir J. https://doi.org/10.1183/13993003.01815-2019

17. Gugala E, Okoh CM, Ghosh S, Moczygemba LR (2022) Pulmonary Health Effects of Electronic Cigarettes: A Scoping Review. Health Promot Pract 23:388–396

18. Li X, Zhang Y, Zhang R, Chen F, Shao L, Zhang L (2022) Association Between E-Cigarettes and Asthma in Adolescents: A Systematic Review and Meta-Analysis. Am J Prev Med 62:953–960

19. Wills TA, Choi K, Pokhrel P, Pagano I (2022) Tests for confounding with cigarette smoking in the association of E-cigarette use with respiratory disorder: 2020 National-Sample Data. Prev Med (Baltim). https://doi.org/10.1016/J.YPMED.2022.107137

20. Palamidas A, Tsikrika S, Katsaounou PA, Vakali S, Gennimata SA, kaltsakas G, Gratziou C, Koulouris N (2017) Acute effects of short term use of ecigarettes on Airways Physiology and Respiratory Symptoms in Smokers with and without Airway Obstructive Diseases and in Healthy non smokers. Tob Prev Cessat. https://doi.org/10.18332/TPC/67799

21. Hedman L, Backman H, Stridsman C, Bosson JA, Lundbäck M, Lindberg A, Rönmark E, Ekerljung L (2018) Association of Electronic Cigarette Use With Smoking Habits, Demographic Factors, and Respiratory Symptoms. JAMA Netw Open 1:e180789

22. Wang JB, Olgin JE, Nah G, Vittinghoff E, Cataldo JK, Pletcher MJ, Marcus GM (2018) Cigarette and e-cigarette dual use and risk of cardiopulmonary symptoms in the Health eHeart Study. PLoS One. https://doi.org/10.1371/JOURNAL.PONE.0198681

23. Bayly JE, Bernat D, Porter L, Choi K (2019) Secondhand Exposure to Aerosols From Electronic Nicotine Delivery Systems and Asthma Exacerbations Among Youth With Asthma. Chest 155:88–93

24. Thirion-Romero I, Pérez-Padilla R, Zabert G, Barrientos-Gutierrez I (2019) RESPIRATORY IMPACT OF ELECTRONIC CIGARETTES AND “LOW-RISK” TOBACCO. Rev Invest Clin 71:17–27

25. Amato L, Cruciani F, Solimini R, Barca A, Pacifici R, Davoli M (2020) [Effects of electronic cigarettes on health: a systematic review of the available evidence]. Recenti Prog Med 111:30–43

26. Underner M, Perriot J, Peiffer G, Jaafari N (2020) [Electronic cigarette use in patients with asthma]. Rev Med Liege 75:613–618

27. Chaffee BW, Barrington-Trimis J, Liu F, Wu R, McConnell R, Krishnan-Sarin S, Leventhal AM, Kong G (2021) E-cigarette use and adverse respiratory symptoms among adolescents and Young adults in the United States. Prev Med (Baltim). https://doi.org/10.1016/J.YPMED.2021.106766

28. Entwistle MR, Valle K, Schweizer D, Cisneros R (2021) Electronic cigarette (e-cigarette) use and frequency of asthma symptoms in adult asthmatics in California. J Asthma 58:1460–1466

29. Polosa R, Morjaria J, Caponnetto P, Caruso M, Strano S, Battaglia E, Russo C (2014) Effect of smoking abstinence and reduction in asthmatic smokers switching to electronic cigarettes: evidence for harm reversal. Int J Environ Res Public Health 11:4965–4977

30. Goniewicz ML, Miller CR, Sutanto E, Li D (2020) How effective are electronic cigarettes for reducing respiratory and cardiovascular risk in smokers? A systematic review. Harm Reduct J. https://doi.org/10.1186/S12954-020-00440-W

31. Solinas A, Paoletti G, Firinu D, Di Pino M, Tusconi M, Mura JF, Del Giacco S, Marongiu F (2020) Vaping effects on asthma: results from a web survey and clinical investigation. Intern Emerg Med 15:663–671

32. Franks AS, Sando K, McBane S (2018) Do Electronic Cigarettes Have a Role in Tobacco Cessation? Pharmacotherapy 38:555–568

33. Bhatnagar A, Payne TJ, Robertson RM (2019) Is There A Role for Electronic Cigarettes in Tobacco Cessation? J Am Heart Assoc. https://doi.org/10.1161/JAHA.119.012742

34. Hernandez ML, Burbank AJ, Alexis NE, Rebuli ME, Hickman ED, Jaspers I, Guidos G (2021) Electronic Cigarettes and Their Impact on Allergic Respiratory Diseases: A Work Group Report of the AAAAI Environmental Exposures and Respiratory Health Committee. J Allergy Clin Immunol Pract 9:1142–1151

35. Kaur G, Pinkston R, McLemore B, Dorsey WC, Batra S (2018) Immunological and toxicological risk assessment of e-cigarettes. Eur Respir Rev. https://doi.org/10.1183/16000617.0119-2017

36. Bozier J, Chivers EK, Chapman DG, Larcombe AN, Bastian NA, Masso-Silva JA, Byun MK, McDonald CF, Crotty Alexander LE, Ween MP (2020) The Evolving Landscape of e-Cigarettes: A Systematic Review of Recent Evidence. Chest 157:1362–1390

37. Gordon T, Karey E, Rebuli ME, Escobar YNH, Jaspers I, Chen LC (2022) E-Cigarette Toxicology. Annu Rev Pharmacol Toxicol 62:301–322

**Supplementary Table 4. The health impact of e-cigarettes/ HnB on COPD outcomes**. **Recent studies (2014-2023) providing the listed Key informative evidence.**

|  | **Key informative evidence** | **Most relevant supporting articles** |
| --- | --- | --- |
| 1 | In e-cigarette users there is an increased incidence of COPD compared to non-users | Wang J 2018 [1]  Perez M 2019 [2]  Wills T 2019 [3]  Amato L 2020 [4]  Barrameda R 2020 [5]  Xie Z 2020 [6]  Bircan E 2021 [7]  Chaffee B 2021 [8]  Kim T 2021 [9]  Antwi G 2022 [10]  Gugala E 2022 [11]  Wills T 2022 [12] |
| 2 | In subjects affected by COPD e-cigarettes use was associated with an increase of symptoms and impairment of lung function | Bowler R 2017 [13]  Wills T 2019 [3]  Barrameda R 2020 [5]  Gugala E 2022 [11]  Wills T 2022 [12] |
| 3 | E-cigarettes (EC) use results in reduced harm and improved outcomes when compared with combustible cigarette (CC) use or dual CC-EC use | Farsalinos K 2014 [14]  Polosa R 2016 [15]  Polosa R 2018 [16]  Ratajczak A 2018 [17]  Goniewicz M 2020 [18]  Polosa R 2020 [19]  Xie Z 2020 [6]  Wilson N 2021 [20]  Gugala E 2022 [11]  Hajat C 2022 [21] |
| 4 | In COPD patients, switch from conventional cigarettes to heated tobacco products can lead to a decrease in exacerbations and improvements in symptoms and physical activity level | Polosa R 2021 [22] |
| 5 | Despite growing evidence to that effect, there is no unanimous consensus on the role of e-cigarettes in helping COPD adults to reduce smoking | Polosa R 2016 [23]  Bowler R 2017 [13]  Franks A 2018 [24]  Polosa R 2018 [16]  Polosa R 2020 [19] |
| 6 | Due to the relatively recent use of vaping, the evolving landscape of e-cigarettes and the paucity of experimental data and consistent evidence, nowadays it is difficult to draw conclusive outcomes regarding toxicological, immunological and clinical impact of e-cigarette aerosols. Moreover, decades of chronic smoking are needed for development of lung diseases such as COPD, so the current knowledge of the effects of e-cigarette is insufficient to determine whether the respiratory health effects of e-cigarette are less than those of combustible tobacco products | Kaisar M 2016 [25]  Kaur G 2018 [26]  Gotts J 2019 [27] |

**REFERENCES**

1. Wang JB, Olgin JE, Nah G, Vittinghoff E, Cataldo JK, Pletcher MJ, Marcus GM (2018) Cigarette and e-cigarette dual use and risk of cardiopulmonary symptoms in the Health eHeart Study. PLoS One. https://doi.org/10.1371/JOURNAL.PONE.0198681

2. Perez MF, Atuegwu NC, Mead EL, Oncken C, Mortensen EM (2019) Adult E-Cigarettes Use Associated with a Self-Reported Diagnosis of COPD. Int J Environ Res Public Health. https://doi.org/10.3390/IJERPH16203938

3. Wills TA, Pagano I, Williams RJ, Tam EK (2019) E-cigarette use and respiratory disorder in an adult sample. Drug Alcohol Depend. https://doi.org/10.1016/j.drugalcdep.2018.10.004

4. Amato L, Cruciani F, Solimini R, Barca A, Pacifici R, Davoli M (2020) [Effects of electronic cigarettes on health: a systematic review of the available evidence]. Recenti Prog Med 111:30–43

5. Barrameda R, Nguyen T, Wong V, Castro G, Rodriguez de la Vega P, Lozano J, Zevallos J (2020) Use of E-Cigarettes and Self-Reported Lung Disease Among US Adults. Public Health Rep 135:785–795

6. Xie Z, Ossip DJ, Rahman I, Li D (2020) Use of Electronic Cigarettes and Self-Reported Chronic Obstructive Pulmonary Disease Diagnosis in Adults. Nicotine Tob Res 22:1155–1161

7. Bircan E, Bezirhan U, Porter A, Fagan P, Orloff M (2021) Electronic cigarette use and its association with asthma, chronic obstructive pulmonary disease (COPD) and asthma-COPD overlap syndrome among never cigarette smokers. Tob Induc Dis 19:1–10

8. Chaffee BW, Barrington-Trimis J, Liu F, Wu R, McConnell R, Krishnan-Sarin S, Leventhal AM, Kong G (2021) E-cigarette use and adverse respiratory symptoms among adolescents and Young adults in the United States. Prev Med (Baltim). https://doi.org/10.1016/J.YPMED.2021.106766

9. Kim SY, Sim S, Choi HG (2017) Active, passive, and electronic cigarette smoking is associated with asthma in adolescents. Sci Rep. https://doi.org/10.1038/S41598-017-17958-Y

10. Antwi GO, Rhodes DL (2022) Association between E-cigarette use and chronic obstructive pulmonary disease in non-asthmatic adults in the USA. J Public Health (Oxf) 44:158–164

11. Gugala E, Okoh CM, Ghosh S, Moczygemba LR (2022) Pulmonary Health Effects of Electronic Cigarettes: A Scoping Review. Health Promot Pract 23:388–396

12. Wills TA, Choi K, Pokhrel P, Pagano I (2022) Tests for confounding with cigarette smoking in the association of E-cigarette use with respiratory disorder: 2020 National-Sample Data. Prev Med (Baltim). https://doi.org/10.1016/J.YPMED.2022.107137

13. Bowler RP, Hansel NN, Jacobson S, et al (2017) Electronic Cigarette Use in US Adults at Risk for or with COPD: Analysis from Two Observational Cohorts. J Gen Intern Med 32:1315–1322

14. Farsalinos KE, Romagna G, Tsiapras D, Kyrzopoulos S, Voudris V (2014) Characteristics, perceived side effects and benefits of electronic cigarette use: a worldwide survey of more than 19,000 consumers. Int J Environ Res Public Health 11:4356–4373

15. Polosa R, Morjaria JB, Caponnetto P, Prosperini U, Russo C, Pennisi A, Bruno CM (2016) Evidence for harm reduction in COPD smokers who switch to electronic cigarettes. Respir Res. https://doi.org/10.1186/S12931-016-0481-X

16. Polosa R, Morjaria JB, Prosperini U, Russo C, Pennisi A, Puleo R, Caruso M, Caponnetto P (2018) Health effects in COPD smokers who switch to electronic cigarettes: a retrospective-prospective 3-year follow-up. Int J Chron Obstruct Pulmon Dis 13:2533–2542

17. Ratajczak A, Feleszko W, Smith DM, Goniewicz M (2018) How close are we to definitively identifying the respiratory health effects of e-cigarettes? Expert Rev Respir Med 12:549–556

18. Goniewicz ML, Miller CR, Sutanto E, Li D (2020) How effective are electronic cigarettes for reducing respiratory and cardiovascular risk in smokers? A systematic review. Harm Reduct J. https://doi.org/10.1186/S12954-020-00440-W

19. Polosa R, Morjaria JB, Prosperini U, Busà B, Pennisi A, Malerba M, Maglia M, Caponnetto P (2020) COPD smokers who switched to e-cigarettes: health outcomes at 5-year follow up. Ther Adv Chronic Dis. https://doi.org/10.1177/2040622320961617

20. Wilson N, Summers JA, Ait Ouakrim D, Hoek J, Edwards R, Blakely T (2021) Improving on estimates of the potential relative harm to health from using modern ENDS (vaping) compared to tobacco smoking. BMC Public Health. https://doi.org/10.1186/S12889-021-12103-X

21. Hajat C, Stein E, Shantikumar S, Niaura R, Ferrara P, Polosa R (2022) A scoping review of studies on the health impact of electronic nicotine delivery systems. Intern Emerg Med 17:241–268

22. Polosa R, Morjaria JB, Prosperini U, Busà B, Pennisi A, Gussoni G, Rust S, Maglia M, Caponnetto P (2021) Health outcomes in COPD smokers using heated tobacco products: a 3-year follow-up. Intern Emerg Med 16:687–696

23. Polosa R, Morjaria JB, Caponnetto P, Caruso M, Campagna D, Amaradio MD, Ciampi G, Russo C, Fisichella A (2016) Persisting Long Term Benefits of Smoking Abstinence and Reduction in Asthmatic Smokers Who Have Switched to Electronic Cigarettes. Discov Med 21:99–108

24. Franks AS, Sando K, McBane S (2018) Do Electronic Cigarettes Have a Role in Tobacco Cessation? Pharmacotherapy 38:555–568

25. Kaisar MA, Prasad S, Liles T, Cucullo L (2016) A decade of e-cigarettes: Limited research & unresolved safety concerns. Toxicology 365:67–75

26. Kaur G, Pinkston R, McLemore B, Dorsey WC, Batra S (2018) Immunological and toxicological risk assessment of e-cigarettes. Eur Respir Rev. https://doi.org/10.1183/16000617.0119-2017

27. Gotts JE, Jordt SE, McConnell R, Tarran R (2019) What are the respiratory effects of e-cigarettes? BMJ. https://doi.org/10.1136/BMJ.L5275
